# Chemical Characterization and Antibacterial Activity of Royal Jelly Against Multidrug-Resistant Pathogens

**DOI:** 10.17113/ftb.64.01.26.9194

**Published:** 2026-02-15

**Authors:** Mirna Mrkonjić Fuka, Irina Tanuwidjaja, Valentina Odorčić, Slaven Jurić, Igor Jerković, Nikolina Udiković-Kolić, Marko Vinceković, Lidija Svečnjak

**Affiliations:** 1 Department Microbiology, Faculty of Agriculture, University of Zagreb, Svetošimunska 25, 10000 Zagreb, Croatia; 2 Department of Chemistry, Faculty of Agriculture, University of Zagreb, Svetošimunska 25, 10000, Zagreb, Croatia; 3 Department of Organic Chemistry, Faculty of Chemistry and Technology, University of Split, Ruđera Boškovića 35, 21000 Split, Croatia; 4 Mediterranean Institute for Life Sciences, University of Split, Meštrovićevo šetalište 45, 21000 Split, Croatia.; 5 Division for Marine and Environmental Research, Ruđer Bošković Institute, Bijenička 54 10000 Zagreb, Croatia; 6 Department of Fisheries, Apiculture, Wildlife Management and Special Zoology, Faculty of Agriculture, University of Zagreb, Svetošimunska 25, 10000 Zagreb, Croatia

**Keywords:** multidrug-resistant bacteria, royal jelly, antibacterial activity, gas chromatography–mass spectrometry (GC-MS), Fourier-transform infrared (FTIR) spectroscopy, antioxidant capacity, bioactive compounds

## Abstract

**Research background:**

Given the known antibacterial properties of royal jelly (RJ), we hypothesize that royal jelly could inhibit priority multidrug-resistant (MDR) bacteria, including different strains of vancomycin-resistant *Enterococcus faecium* (VRE), methicillin-resistant *Staphylococcus aureus* (MRSA), carbapenem-resistant *Klebsiella pneumoniae* (CRKP) and *Acinetobacter baumannii* (CRAB). We further propose that the antibacterial efficacy of royal jelly may be influenced by its chemical composition and by inter- and intraspecies variability among MDR pathogens.

**Experimental approach:**

Royal jelly samples were collected from five beekeepers (RJ1–RJ5) in the Mediterranean and continental regions of Croatia. Chemical profiling was conducted using solid-phase microextraction gas chromatography–mass spectrometry (HS-SPME/GC-MS) and Fourier-transform infrared (FTIR) spectroscopy, together with separate assays to measure antioxidant capacity (ABTS) and quantify the content of bioactive compounds. Antibacterial activity was assessed by agar well diffusion assay and by determining the minimum inhibitory concentration (MIC) and minimum bactericidal concentration (MBC) against 20 MDR strains of VRE, MRSA, CRKP and CRAB, selected from 85 isolates using repetitive sequence-based PCR (rep-PCR) genotyping. MDR status was confirmed by standard susceptibility testing.

**Results and conclusions:**

All royal jelly samples showed strong antioxidant activity and high amounts of bioactive compounds, with RJ1 consistently exhibiting the highest contents of ABTS, polyphenols, flavonoids and proteins. FTIR analysis revealed variations in carbohydrate and lipid composition among samples, while protein content remained relatively uniform, and indicated the highest mass fractions of sugars, lipids and proteins in RJ1. GC-MS identified octanoic acid (48.09–83.07 %) as the predominant volatile compound, especially abundant in RJ1 and RJ4. Despite some variability in chemical profiles, both chemical composition and antibacterial activity were comparable between samples from the Mediterranean and continental regions. All royal jelly samples inhibited MDR bacteria, suggesting a potential synergistic effect of crude royal jellies, with inhibition zones ranging from 11.8 (CRKP) to 16.8 mm (MRSA). *A. baumannii* was most susceptible (MIC/MBC=27.2 µg/mL), while *E. faecium* was the most resistant (MIC=96.6 µg/mL, MBC=126.4 µg/mL). Beyond interspecies differences, pronounced strain-level variability in antibacterial response was also observed.

**Novelty and scientific contribution:**

This is the first study to simultaneously evaluate the antibacterial activity of royal jelly against multiple strains of clinically relevant MDR pathogens alongside comprehensive chemical profiling. Importantly, it reveals for the first time that the efficacy of royal jelly varies not only between species but also among strains within the same species, emphasizing the need to consider strain-level differences in future assessments.

## INTRODUCTION

Multidrug-resistant (MDR) bacteria are deadly pathogenic microorganisms that pose a serious threat to human health. In 2019 alone, an estimated 6.27 million people died from infections caused by antibiotic-resistant pathogens, and the World Health Organization (WHO) warns that this number could continue to rise (*1*). MDR bacteria are defined by an increased level of acquired resistance to multiple classes of antibiotics and are involved in various life-threatening healthcare-associated infections. In the past, these antibiotic-resistant bacteria were rare and limited to nosocomial infections, but today they are very common. In addition to hospitals, they are frequently found in environmental samples, including soil, food (such as vegetables, fruit and animal products), water, plants and sewage (*2*). In 2024, WHO revised the Bacterial Priority Pathogens List (BPPL), which includes bacterial pathogens of public health importance (*1*). In this updated BPPL, Gram-negative bacterial pathogens retain their critical status. Carbapenem-resistant *Acinetobacter baumannii* (CRAB), carbapenem-resistant Enterobacterales (CRE) and third-generation cephalosporin-resistant Enterobacterales (3GCRE) received the highest scores, confirming their inclusion in the critical priority category. Methicillin-resistant *Staphylococcus aureus* (MRSA) and vancomycin-resistant *Enterococcus faecium* (VRE) remain in the high-priority pathogen category. Infections caused by both categories of priority pathogens are extremely difficult to treat, as they not only exhibit multidrug resistance but also show resistance to last-resort antibiotics, including carbapenems (*1*). This severely restricts treatment options and leads to a longer duration of illness, a higher burden on the healthcare system and increased mortality (*2*). The global rise of antibiotic resistance has intensified research into alternative therapeutic strategies against MDR pathogens, with particular interest in natural bioactive compounds such as royal jelly (RJ).

Royal jelly is a yellowish, acidic secretion produced by the hypopharyngeal and mandibular glands of honey bees (*Apis mellifera* L.) (worker bees) for the nourishment of the brood (larvae) and the queen bee. It is composed mainly of water, proteins, sugars and lipids and is highly sensitive to heat and light (*3*, *4*). In recent years, the use of royal jelly has increased due to its beneficial properties and health-promoting effects, with great potential in medical and pharmaceutical applications, cosmetics, and as a functional food. The antibacterial properties of royal jelly against both Gram-positive and Gram-negative bacteria have been well documented (*3*), with a stronger inhibitory effect observed against Gram-positive pathogens (*5*). In addition to its antibacterial properties, royal jelly also exhibits anti-inflammatory and immune-stimulating effects (*5*). The antibacterial activity of royal jelly is primarily attributed to the protein royalisin, major royal jelly proteins (MRJPs), jelleines I–III, and the fatty acid 10-hydroxy-2-decenoic acid (10-HDA) (*3*). The overall antibacterial effect of whole royal jelly remains significant, as its complex composition may contribute synergistically to its bioactivity. Notably, the antibacterial potency of royal jelly is influenced by several factors, including the geographical origin, the genetic and physiological characteristics of the bee colony and the botanical composition of the harvested plant species (*3*). Despite the proven antibacterial effect, the number of studies of royal jelly against priority MDR bacterial pathogens is very low. Studies have shown that royal jelly has antibacterial activity against methicillin-resistant *S. aureus* (MRSA; MIC=37.5-75 mg/mL) (*5*), and the effect is highly dependent on the royal jelly sample used. However, there are no data on the effect of crude royal jelly on other priority pathogens such as carbapenem-resistant *A. baumannii* (CRAB), Enterobacterales (CRE) or vancomycin-resistant *E. faecium* (VRE). Moreover, to determine the influence of an active compound on a particular bacterial species, intraspecies variability should be considered. The importance of variability within species has been particularly well studied in the context of pathogenicity, human microbiome (*6*) and environmental samples (*7*). However, the effect of royal jelly on different MDR strains within the same species has not been evaluated.

Therefore, this study aims to investigate the efficacy of royal jelly against genetically diverse strains of priority MDR pathogens, including both Gram-positive (vancomycin-resistant *E. faecium* and methicillin-resistant *S. aureus*) and Gram-negative (carbapenem-resistant *K. pneumoniae* and carbapenem-resistant *A. baumannii*) clinical and environmental isolates, complemented by comprehensive chemical characterization of royal jelly samples. To achieve this, royal jelly samples were collected from five beekeepers across two geographic regions of Croatia, continental and Mediterranean. Antibacterial activity was assessed using the agar well diffusion method followed by determination of minimum inhibitory concentration (MIC) and minimum bactericidal concentration (MBC). Chemical profiles were obtained by headspace solid-phase microextraction (HS-SPME) followed by gas chromatography–mass spectrometry (GC-MS) analysis and Fourier-transform infrared (FTIR) spectroscopy. Bioactivity assessment included evaluation of antioxidant capacity and quantification of bioactive compound content. To the best of our knowledge, this is the first comprehensive study to simultaneously investigate the antibacterial effects of royal jelly against different strains of priority MDR pathogens alongside detailed chemical profiling of the samples.

## MATERIALS AND METHODS

### Sampling

Royal jelly samples were collected in Croatia during the 2022 production season, directly from beekeepers maintaining colonies of Carniolan honey bees (*Apis mellifera carnica*, Pollmann, 1879). Two samples, RJ1 and RJ2, were obtained from Konšćica, Zagreb County, and RJ3 from Petrovsko, Zagorje County, both located in the continental part of Croatia. The remaining two samples, RJ4 and RJ5, originated from farms near Obrovac Sinjski, Dalmatia County, in the Mediterranean region of Croatia. To preserve their integrity, all royal jelly samples were stored in dark containers and frozen at -20 °C immediately after collection. They were transported to the laboratory in a frozen state and kept at -20 °C until analysis.

### Determination of antioxidant capacity, total polyphenolic content and total flavonoid content

A total of 0.5 g of royal jelly was thoroughly homogenized with 5 mL of distilled water. The resulting suspensions were then filtered using Whatman No. 4 filter paper (Sigma-Aldrich, Merck, St. Louis, MO, USA) (*8*). The results obtained were calculated based on a 100 g royal jelly sample.

The 2,2’-azino-bis (3-ethylbenzothiazoline-6-sulfonic acid) (ABTS) (Sigma-Aldrich, Merck) was used to measure the antioxidant potential of the royal jelly samples according to the procedure of Re *et al*. (*9*). The data obtained are expressed as μmol Trolox equivalents per 100 g of royal jelly.

A modified Folin-Ciocalteu method (*10*) was used to determine the total polyphenolic content (TPC). Briefly, a volume of 0.1 mL of royal jelly samples was mixed with 7.9 mL of distilled water. Then, 0.5 mL of Folin-Ciocalteu reagent (Sigma-Aldrich, Merck) (diluted with distilled water to 1:2) and 1.5 mL of 20 % Na_2_CO_3_ (Sigma-Aldrich, Merck) were added. After 2 h, absorbance was measured at 765 nm using a UV-Vis spectrophotometer (UV-1700; Shimadzu, Kyoto, Japan). The calibration curve was established using gallic acid (Sigma-Aldrich, Merck) and results were expressed as mg gallic acid equivalents (GAE) per 100 g of royal jelly.

The total flavonoid content (TFC) was determined using a previously reported method (*11*). A volume of 1 mL of the royal jelly was added to a 10-mL volumetric flask containing 4 mL of distilled water. Next, 300 μL of NaNO_2_ (Sigma-Aldrich, Merck) solution (0.5 g/L) was added to the suspension. After 5 min, a volume of 300 μL of AlCl_3_ (Sigma-Aldrich, Merck) (1 g/L) was added. Six minutes later, 2 mL of NaOH (Sigma-Aldrich, Merck) (1 mol/L) were added to the mixture. The final volume was adjusted to 10 mL with distilled water. Absorbance was measured at 360 nm against a blank (distilled water) with a UV-Vis spectrophotometer (UV-1700; Shimadzu). A calibration curve was generated using quercetin (Sigma-Aldrich, Merck) standard, and the results were expressed as mg of quercetin equivalents (QE) per 100 g of royal jelly.

Total proteins (TP) were determined using the Lowry method (*12*). Two reagents were prepared: reagent A (2 % (*m*/*V*) Na_2_CO_3_ (Sigma-Aldrich, Merck) in 0.1 mol/L NaOH (Sigma-Aldrich, Merck)) and reagent B (0.5 % (*m*/*V*) CuSO_4_·5H_2_O (Sigma-Aldrich, Merck) in 1 % (*m*/*V*) KNaC_4_H_4_O_6_·4H_2_O (Sigma-Aldrich, Merck)). Reagent A (50 mL) was mixed with reagent B (1 mL) to obtain reagent C. A volume of 0.2 mL of Folin-Ciocalteu reagent was added to 0.4 mL of water-solubilized royal jelly in a test tube. Finally, 2 mL of reagent C were added and 50 min after the start of the chemical reaction, absorbance was measured at 740 nm against a blank using a UV-1700 spectrophotometer (Shimadzu). A bovine serum albumin (Sigma-Aldrich, Merck) standard was used for the calibration curve and the results were expressed as mg of bovine serum albumin equivalents (BSAE) per 100 g of royal jelly.

### Fourier transform infrared spectroscopy-attenuated total reflectance

Samples of royal jelly were analysed by Fourier transform infrared spectroscopy (FTIR) coupled with the attenuated total reflectance (ATR) technique, following the general instrumentation requirements and methodology of Hu *et al*. (*13*), modified for the acquisition of infrared spectra of royal jelly samples in their genuine form (as obtained). FTIR-ATR spectra of the royal jelly samples were recorded using Cary 660 Fourier transform mid-infrared spectrometer (Agilent Technologies, Santa Clara, CA, USA) coupled with a single-reflection diamond Golden Gate ATR accessory (Specac Ltd, Orpington, UK). The FTIR-ATR spectra of royal jelly were acquired in the mid-infrared region (4000–400 cm^-1^) with a nominal recording resolution of 4 cm^-1^. Two replicate spectra of each royal jelly sample were recorded using different aliquots (32 scans were collected for each spectrum). Spectra of royal jelly samples were recorded at room temperature ((24±2) °C). Raw spectral data were stored and pre-analysed using Resolutions Pro v. 5.3.0 (*14*) FTIR software (Agilent Technologies), while further qualitative spectral data analysis was performed using Spectragryph optical spectroscopy software v. 1.2.15 (*15*) and Origin v. 8.1 (*16*).

### Headspace solid-phase microextraction and gas chromatography coupled with mass spectrometry

A mass of 0.2 g of each sample was placed separately in a 20-mL headspace vial, sealed with PTFE-silicone septum, and the headspace was extracted using a manual holder (Supelco Co., Bellefonte, PA, USA) with two fibres: divinylbenzene/carboxene/polydimethylsiloxane (DVB/CAR/PDMS; 50 μm (DVB layer) and 30 μm (CAR/PDMS layer); *l*(fibre)=1 cm) and polydimethylsiloxane/divinylbenzene (PDMS/DVB; 65 μm (PDMS/DVB), *l*(fibre)=1 cm), both purchased from Supelco Co. The fibres were conditioned according to Supelco instructions. The sample was equilibrated for 15 min at 60 °C and then extracted for 40 min. Thermal desorption of the fibre was performed directly into the GC column for 7 min at 250 °C.

The gas chromatography-mass spectrometry (GC-MS) analysis was conducted using an Agilent Technologies gas chromatograph model 7820A equipped with a mass selective detector (MSD) model 5977E (Agilent Technologies) and an HP-5MS capillary column (5 % phenylmethylpolysiloxane; Agilent Technologies). The GC conditions were as follows: the oven temperature was held isothermal at 70 °C for 2 min, then increased from 70 to 200 °C at 3 °C/min, and held isothermal at 200 °C for 15 min; the carrier gas was He (1.0 mL/min). The MSD (EI mode) was operated at 70 eV with a mass range of 30-300 amu. The GC-MS analyses were performed in triplicate, and mean values of the area percentages were determined. The identification of the compounds involved comparison of their mass spectra with the mass spectral libraries Wiley 9 (Wiley, New York, NY, USA) and NIST 14 (National Institute of Standards and Technology, Gaithersburg, MD, USA), with the selectivity index (the highest probability of the experimental mass spectrum matching the reference library spectrum) set up to 95 %. Additionally, retention indices (RI), calculated based on the retention times of C_9_-C_25_ *n*-alkanes for each compound, were compared with those reported in the literature (National Institute of Standards and Technology).

### Origin of multidrug-resistant bacterial isolates

Bacterial isolates of *K. pneumoniae* (*N*=40) and *E. faecium* (*N*=9) used in this study were previously isolated from treated municipal and untreated hospital wastewater in Zagreb, Croatia, as described by Puljko *et al.* (*17*), and were kindly provided by the Laboratory for Environmental Microbiology and Biotechnology, Ruđer Bošković Institute. *Acinetobacter baumannii* (*N*=10), *Staphylococcus aureus* (*N*=15) and *E. faecium* (*N*=11) were isolated from clinical specimens and kindly provided by the Clinic for Infectious Diseases “Dr. Fran Mihaljević”, Zagreb, Croatia. All environmental isolates were identified by matrix assisted laser desorption ionization-time of flight (MALDI-TOF) MS (Bruker Daltonik, Bremen, Germany), while clinical isolates were identified by the VITEK 2 System (bioMérieux, Marcy-l'Étoile, France).

### Extraction of DNA and genotyping of bacterial isolates

Genomic DNA was extracted from all bacterial isolates (*N*=85) using the Wizard Genomic DNA Purification Kit (Promega, Madison, WI, USA) according to the manufacturer’s instructions. Genotyping was performed using repetitive element PCR (rep-PCR) with the (GTG)_5_ primer (Microsynth Austria, Wien, Austria) (*18*). The obtained rep-PCR patterns were analysed in BioNumerics 7.6.1 software (*19*), and the genetic similarity of the isolates was calculated using the Dice coefficient. Isolates were then clustered by the Unweighted Pair Group Method with Arithmetic Average (UPGMA) and dendrograms were created with a 1.0 % tolerance level and 0.5 % optimization. Based on the obtained profiles, representative isolates (*N*=5) were selected from each group for further analysis.

### Antibiotic susceptibility testing

All representative strains were screened for antibiotic susceptibility using the Kirby-Bauer disc diffusion method to confirm their MDR profile. A McFarland standard 0.5, containing approx. 1.5·10^8^ CFU/mL, was achieved by adding individual colonies of each strain to a sterile 0.85 % saline solution (DEN-1 densitometer; Biosan, Riga, Latvia). Mueller-Hinton (MH) agar plates (Biolife, Monza, Italy) were then inoculated with a 1.5·10^8^ CFU/mL bacterial suspension, followed by the addition of antibiotic discs (BD BBL™ Sensi-Disc™; Becton Dickinson and Company, Franklin Lakes, NJ, USA). After 24 h of incubation at 37 °C, zones of inhibition were measured and interpreted according to Clinical and Laboratory Standards Institute (CLSI) guidelines (*20*, *21*). Antibiotics were selected based on CLSI recommendations for each species and are listed in Table S1.

### Screening of antibacterial activity using the agar well diffusion method

Antibacterial activity was first evaluated using a modified Kirby-Bauer agar well diffusion method (*20*). For each royal jelly sample, a working solution (1 g/mL) was prepared by dissolving 6 g of royal jelly in 6 mL of sterile MH broth (Biolife) and thoroughly mixing with a vortex mixer. To protect the royal jelly from light degradation, the solutions were wrapped in aluminium foil immediately after preparation. MH agar plates were inoculated with a bacterial suspension adjusted to 1.5·10^8^ CFU/mL, as described above. Wells of 9 mm in diameter were aseptically punched into the agar using a sterile cork borer. Each well was filled with 0.2 mL of the royal jelly working solution, applied in duplicate. As a negative control, 0.2 mL of dimethyl sulfoxide (DMSO; Sigma-Aldrich, Merck) was used. The plates were then incubated at 37 °C for 24 h, after which the zones of inhibition, including the diameter of the well, were measured.

### Determining MIC and MBC

MIC was determined using the microdilution method with the addition of resazurin (Santa Cruz Biotechnology, Dallas, TX, USA) (resazurin microplate assay, REMA (*22*, *23*)). This method involves the serial dilution of royal jelly in a 1:2 ratio in MH broth. Testing was performed in 96-well microtiter plates, where royal jelly samples were diluted aseptically (7.81, 15.63, 31.25, 62.50, 125.00, 250.00 and 500.00 mg/mL) in MH broth.

Each bacterial inoculum (1.5·10^8^ CFU/mL) was diluted in sterile saline solution to 1.5·10^6^ CFU/mL and subsequently added to microtiter wells to reach 1.5·10^5^ CFU/mL per well. A control included MH broth with bacterial inoculum but without royal jelly. After inoculation, 15 µL of resazurin (0.02 % solution) were added to each well as a bacterial growth indicator. Plates were incubated at 37 °C under constant shaking (90 rpm) for 24 h. A blue to pink colour change indicated bacterial growth. The lowest royal jelly concentration at which no colour change occurred (*i.e.* no visible growth) was recorded as the MIC. For MBC, contents of each well were transferred onto brain heart infusion (BHI) agar plates (Biolife) in quadruplicate and incubated at 37 °C for 24 h, after which bacterial colonies were counted. The percentage of dead cells following royal jelly treatment was calculated according to the following equation:


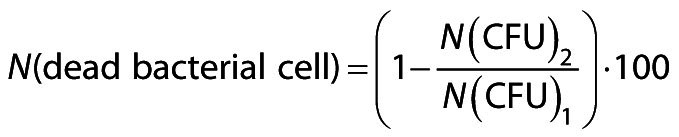
 /1/

where *N*(CFU)_1_ represents the number of bacteria initially added to the wells, and *N*(CFU)_2_ represents the number of bacteria that survived royal jelly treatment.

To confirm the *N*(CFU)_1_, an additional serial microdilution was performed in a 96-well plate using a sterile 0.85 % saline solution. The prepared bacterial inocula (1.5·10^6^ CFU/mL) were aseptically diluted 1:10 from row A to row H, and the contents were transferred onto BHI agar in quadruplicate. After incubation at 37 °C for 24 h, CFU/mL was calculated. Finally, the royal jelly concentration at which more than 99.9 % of bacterial cells were killed was recorded as the MBC, while the concentration at which more than 99.5 % of bacterial cells were inhibited was recorded as the MIC (*24*).

### Statistical analysis

All data are shown as mean values with standard deviations. Based on the normal distribution and homogeneous variance of the data, significant differences in antioxidant activity, TPC, TFC and TP were assessed by ANOVA, and between-group differences were identified by *post hoc t*-tests, with p-values adjusted using the Bonferroni correction. For data with a non-normal distribution and heterogeneous variance, statistically significant differences in inhibition zones, MIC and MBC values were assessed using the Kruskal-Wallis test. To determine statistically significant differences between groups, multiple pairwise comparisons were performed using *post hoc* Dunn’s test, with p-values adjusted for multiple comparisons using the Bonferroni correction. For all analyses, differences with p<0.05 were considered statistically significant. All statistical analyses were performed in the R environment v. 3.0.2 (*25*).

## RESULTS AND DISCUSSION

### Antioxidant activity, total polyphenolic content, total flavonoid content and total proteins in royal jelly

Significant differences in antioxidant activity were observed among the tested royal jellies (Table 1), regardless of the region (Mediterranean or continental). Samples RJ4 and RJ5, collected from the Mediterranean region, had similar ABTS values and did not differ significantly from the other tested royal jellies. Notably, significant differences were found in samples from continental areas (RJ1 and RJ2). The antioxidant activity of royal jellies can be attributed to the wide range of bioactive compounds they contain. For example, the major fatty acid with antioxidant activity in royal jelly is 10-hydroxydecanoic acid (10-HDA), which is present only in royal jelly and not in other natural raw materials or apiculture products (*26*). Furthermore, since polyphenolic compounds are generally recognized as key contributors to antioxidant activity, it is likely that they are also responsible for the antioxidant activity of our royal jelly samples.

**Table 1:** Antioxidant capacity (ABTS) and bioactive compounds as total polyphenolic compounds (TPC), total flavonoid content (TFC) and total proteins (TP) content in royal jelly samples (RJ1-RJ5)

Royal jelly	Antioxidant capacity	Bioactive compound		
	ABTS as *b*(TE)/(μmol/100 g)	TPC as *w*(GAE)/(mg/100 g)	TFC as *w*(QE)/(mg/100 g)	TP as *w*(BSAE)/(g/100 g)
RJ1	(353±8.)^a^	(416±7)^ab^	(18.2±0.2)^a^	(16.5±0.1)^a^
RJ2	(285±6)^b^	(356±15)^a^	(16.0±0.3)^b^	(12.80±0.07)^b^
RJ3	(293±5)^b^	(402±11)^ab^	(14.4±0.2)^c^	(13.4±0.2)^b^
RJ4	(326±11)^ab^	(465±13)^b^	(15.7±0.3)^bc^	(15.6±0.2)^c^
RJ5	(305±11)^ab^	(406±7)^b^	(16.47±0.08)^b^	(14.7±0.2)^d^
Origin				
Continental	(310±33)^a^	(391±30)^a^	(16.2±1.7)^a^	(14.2±1.7)^a^
Dalmatia	(316±17)^a^	(436±24)^b^	(16.1±0.5)^a^	(15.1±0.5)^a^

The results are shown as a mean value±standard deviation. Different letters in superscript in the same column indicate significant differences in antioxidant capacity, TPC, TFC and TP based on *t*-tests with Bonferroni adjusted p-values (p<0.05). TE=Trolox equivalents, GAE=gallic acid equivalents, QE=quercetin equivalents, BSAE=bovine serum albumin equivalents

The TPC, expressed as GAE, in royal jelly ranged from 356 to 465 mg/100 g, with the lowest value recorded at the continental location Konšćica (RJ2), which was significantly lower than those from Dalmatia County. Similar values, expressed as GAE, have been reported for six different royal jelly samples collected from Morocco, Portugal and Spain, ranging from 300 to 900 mg/100 g (*27*). Using the same preparation method, Pavel *et al*. (*8*) reported much higher TPC values, expressed as GAE, in Romanian royal jelly, with average values from 2325 mg/100 g for commercial to 2349 mg/100 g for locally produced royal jelly. Nabas *et al*. (*28*) also reported similar and higher values, with an average of 2330 mg/100 g. Čeksterytė *et al*. (*29*) reported lower values of 1070 mg/100 g for Lithuanian royal jelly solubilized in methanol/water. Harvesting time has been shown to significantly affect the antioxidant compound content of royal jelly, including TPC (*30*). Özkök and Silici (*31*) reported a significantly lower TPC value (59.16 mg/100 g) in Turkish royal jelly, likely due to the use of pure methanol as a solvent. This highlights the impact of solvent choice, with water (or a methanol/water mixture) appearing more effective for TPC solubilization. Overall, TPC data in royal jelly remain scarce and show marked diversity.

Regarding total flavonoids, expressed as QE, in this study they ranged from 14.4 to 18.2 mg/100 g, consistent with values reported by El-Guendouz *et al*. (*27*), who found 10-50 mg/100 g in royal jelly samples from Morocco, Portugal and Spain. In contrast, Nabas *et al*. (*28*) reported significantly higher values expressed as rutin (128 mg/100 g) in Jordanian royal jelly, which also showed significantly higher TPC values, as mentioned above.

The highest total protein content in this study was recorded for RJ1 (16.5 %), significantly exceeding all other royal jelly samples, while the lowest was in RJ2 (12.80 %), despite both being from the same area. These values are in line with literature reports, which typically range from 11.4 to 15.8 % (*26*). For example, in 19 local Romanian royal jelly samples, protein content ranged from 9.58 to 16.38 % using the Lowry method and from 8.15 to 17.73 % according to the Bradford method (*8*). Nabas *et al*. (*28*) reported 13.15 % total proteins in royal jellies produced in Jordan. The protein content reported in the literature is variable and depends on the method of protein determination, but overall, reported protein values tend to be more consistent and reliable than TPC or TFC. Generally, literature on royal jelly is scarce and values for compounds like polyphenolics and flavonoids, as well as antioxidant activity, are largely underreported. One of the aims of this work is to expand the knowledge of bioactive compounds and antioxidant activity in royal jellies collected from Croatian continental and Mediterranean sites. The observed variability in values among samples from the same area indicates that, beyond geographic origin, other parameters likely influence the quality of royal jelly.

### Chemical characterisation of royal jelly by FTIR-ATR spectroscopy

**Fig. 1:**
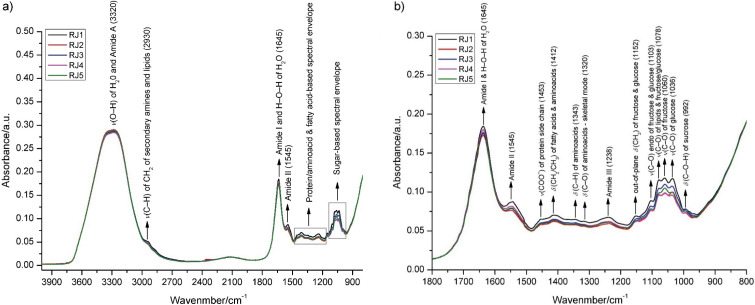
FTIR-ATR spectra of royal jelly samples (*N*=5; RJ1–RJ5) representing comparative spectral features with identification of major underlying molecular vibrations: a) whole spectral region (4000–800 cm^-1^), b) spectral range between 1800 and 800 cm^-1^ with emphasized fingerprint region (1500–800 cm^-1^). *ν*=stretching vibration, *δ*=deformation vibration (bending)

Fig. 1 shows that the FTIR-ATR spectra of fresh royal jelly samples representing comparative spectral features with identified major underlying molecular vibrations reflect the overall chemical composition of analysed royal jelly samples. According to Sabatini *et al*. (*4*), fresh royal jelly typically consists of 60–70 % water, 9–18 % proteins, 7–18 % carbohydrates (*i.e.* fructose, glucose and sucrose content), 3–8 % lipids, >1.4 % 10-hydroxy-2-decenoic acid (10-HDA), and 0.8–3 % ash. Fructose, glucose and sucrose are the predominant sugars in royal jelly, with average contents of 3–13, 4–8 and 0.5–2 %, respectively. As royal jelly is a complex biological matrix comprising water, major macromolecules (proteins, carbohydrates and lipids), and unique compounds such as 10-HDA and antimicrobial peptides (*4*), its FTIR-ATR spectrum shows a wide variety of absorption bands arising from the molecular vibrations of these constituents.

As shown in Fig. 1a, the most intense absorption band in the royal jelly spectra, with an absorption maximum at 3320 cm^-1^, is attributed to the stretching vibrations of the hydroxyl groups (O–H) of water and carbohydrates (CHO) (*32*-*34*), as well as the N–H stretching vibrations of proteins, known as amide A band (*33*, *35*). These overlapping effects occur because the amide A band of proteins typically absorbs IR radiation in the spectral range of 3600–3000 cm^-1^ (similar to water and CHO), but it can be assumed that the generally less intense amide A band is overlapped by the more intense vibrations of the O–H groups of water and CHO. The most intense absorption band at 3320 cm^-1^ is followed by a low-intensity IR signal at 2930 cm^-1^. According to Tarantilis *et al*. (*36*), a low-intensity IR signal observed in the royal jelly spectrum at 2930 cm^-1^ corresponds to the C–H stretching vibrations of the -CH_2_ groups and secondary amines. However, it can be assumed that this band corresponds to the stretching vibrations of the CH_2_ groups of both amines (and/or proteins) and lipids, primarily fatty acids, as they are the most abundant component of the lipid fraction in royal jelly (*4*, *36*) and typically absorb IR radiation strongly in this region (*33*, *37*, *38*); this is represented by the asymmetric and symmetric stretching vibrations of the CH_2_ groups of lipids (aliphatic chains).

The spectral region between 1800 and 800 cm^-1^ contains several absorption bands associated with various royal jelly constituents, *i.e.* proteins, lipids and carbohydrates (primarily glucose, fructose and sucrose, as the predominant sugars in royal jelly), The most prominent band in this region is a medium-intensity vibration at 1645 cm^-1^, assigned to C=O and C–N stretching vibrations (amide I band) of royal jelly proteins. Amide I band primarily consists of stretching vibrations of the C=O group (70–85 %) and, to a lesser extent, the C–N group (10–20 %) (*33*, *35*). In addition to being typical for protein β-sheet structures, this band position is also characteristic of the well-known molecular vibration of water (H–O–H deformation), thus reflecting an overlapping spectral effect related to these royal jelly constituents. Similar findings were reported by Tarantilis *et al*. (*36*). A peak observed at 1545 cm^-1^ is attributed to amide II band of proteins, which comprises N–H bending and C–N stretching vibrations (*33*, *35*, *36*).

A series of less intense absorption bands in the spectral region from 1480 to 1135 cm^-1^ (at 1453, 1412, 1343, 1320 and 1238 cm^-1^) are primarily attributed to vibrations of the functional groups of proteins and amino acids, and to a lesser extent, fatty acids (*33*, *37*), as shown in Fig. 1b. The signal at 1238 cm^-1^ represents a band position characteristic of amide III of proteins, which comprises 30 % N–H bending, 30 % C–N stretching, 10 % C–O stretching, and 10 % of O=C–N bending vibrations (*33*, *35*). The overlapping of CH_2_ bending of fatty acids and CH_3_ bending of amino acids (both exhibiting weak signals in this region) is likely for the signal at 1412 cm^-1^. In the spectral region from 1160 to 965 cm^-1^ a medium-intensity band with an absorption maximum at 1078 cm^-1^ can be attributed to the C–O stretching vibrations of lipids (although it overlaps with C–O stretching of fructose and glucose, as explained below), while the stretching vibrations of the C–O bonds of fructose and glucose are represented at 1060 and 1036 cm^-1^, respectively (*32*). An absorption band at 992 cm^-1^ is assigned to sucrose-specific C–O–H bending or ring vibration (*34*). Sugars are further represented by an absorption band at 1152 cm^-1^, which is attributed to out-of-plane CH_2_ bending vibration (wagging) of both fructose and glucose, while bands at 1103 and 1078 cm^-1^ can be assigned to C–O endocyclic stretching vibrations of these monosaccharides (*32*, *33*).

As shown in Fig. 1b, with the emphasized fingerprint region (1500–800 cm^-1^), the most prominent differences in the composition of the analysed royal jelly samples were related to different proportions of carbohydrates and lipids, while the protein fraction and water content were found to be less variable components. Based on the spectral data, RJ1 was observed to contain higher amounts of predominant sugars (fructose, glucose and sucrose), lipids and proteins, RJ2 and RJ4 showed similar spectral features, reflecting the lowest amounts of these constituents, while RJ3 and RJ5 samples revealed medium amounts. The most variable segment of the analysed royal jelly spectra was the predominantly sugar-based spectral envelope (from 1150 to 950 cm^-1^), reflecting various proportions of fructose, glucose and sucrose, as emphasized in Fig. 1b.

### The headspace composition of royal jelly

The headspace composition of royal jelly samples is shown in Table 2. Overall, six compounds were identified by HS-SPME/GC-MS using two fibres. The predominant headspace compound was octanoic acid (48.09–83.07 % with the PDMS/DVB fibre, and 52.30–80.55 % with the DVB/CAR/PDMS fibre). It was the most abundant in the headspace of samples RJ1 and RJ4, while its abundance was the lowest in sample RJ5. The presence of octanoic acid in the volatile fraction of royal jelly was previously found by Boch *et al*. (*39*). A more detailed study using headspace solid-phase microextraction (HS-SPME) followed by diethyl ether and methanol extraction (the extracts were silanized) identified 185 organic compounds (by GC-MS) from 17 samples of royal jelly (*39*). HS-SPME/GC–MS analysis of fresh royal jelly detected 25 compounds (*40*), with octanoic acid as the major aliphatic acid, while the most abundant carbonyls were: heptan-2-one, acetone, nonan-2-one and benzaldehyde. Therefore, the similarity with present results is observable and the differences can be attributed to the much higher amount of royal jelly samples (1.5–2 g) and to the differences in the origin of royal jelly. Octanoic acid was quantified as the major volatile component of royal jelly (113 to 252 μg/g) and its mass fraction is much lower in drone and worker larval food (3.2–7.6 and 2.1–7.3 μg/g, respectively), as determined by extraction with diethyl ether after quantitative GC-MS analysis of the trimethylsilyl derivative (*41*). Boch *et al*. (*39*) suggested a possible biological role for this compound. Octanoic acid, at a mass fraction similar to that found in royal jelly, was as repellent to *Varroa destructor* (both under lab and field conditions) as royal jelly itself (*41*). In addition, octanoic acid exhibited antimicrobial activity against oral microorganisms (*42*) and showed significant anti-*Candida* activity, which could contribute to the observed antibacterial activity against MDR pathogens in the present research.

**Table 2:** Headspace compounds of royal jelly (RJ) samples extracted by headspace solid-phase microextraction (HS-SPME) with two fibres and analysed by gas chromatography and mass spectrometry (GC-MS)

Compound	RI	PDMS/DVB fibre *A*_GC_/%					DVB/CAR/PDMS fibre *A*_GC_/%				
		RJ1	RJ2	RJ3	RJ4	RJ5	RJ1	RJ2	RJ3	RJ4	RJ5
Heptan-2-one	<900	2.93	3.36	10.22	2.47	11.60	1.06	1.39	6.57	2.20	6.74
Benzaldehyde	950	N.D.	N.D.	2.88	3.31	N.D.	2.51	3.38	8.15	9.60	4.46
2-Metoxyphenol	1094	N.D.	3.08	N.D.	N.D.	N.D.	N.D.	N.D.	N.D.	N.D.	N.D.
Nonan-2-one	1095	1.19	3.06	1.57	3.64	22.31	1.08	4.20	4.66	5.79	19.20
Octanoic acid	1183	83.07	69.82	66.43	73.53	48.09	80.55	70.37	65.22	68.33	52.30
2-Methoxy-4-methylphenol (*p*-creosol)	1196	N.D.	2.99	N.D.	0.54	N.D.	N.D.	0.85	N.D.	0.27	N.D.

N.D.=not detected, RI=retention index, standard deviation among the triplicates was <8 %, PDMS/DVB=polydimethylsiloxane/divinylbenzene copolymer, CAR=carboxen, *A*_GC_=area of GC peaks

Two other lower aliphatic ketones were found in minor abundance (Table 2): heptan-2-one (2.93–11.60 % with PDMS/DVB fibre, and 1.06–6.74 % with DVB/CAR/PDMS fibre) and nonan-2-one (1.19–22.31 % with PDMS/DVB fibre, and 1.08–19.20 % with DVB/CAR/PDMS fibre). Heptan-2-one and nonan-2-one were the most abundant in sample RJ5. Heptan-2-one is produced by bee mandibular glands (*43*) and acts as a mild anxiety pheromone (*44*). The repellent activity of heptan-2-one in royal jelly from queen cells can be directed against *Varroa destructor* (*45*) and it can also enhance a repellent action of octanoic acid. Among carbonyl compounds, benzaldehyde was detected in all samples, with higher abundance only by DVB/CAR/PDMS fibre (2.51–9.60 %).

### Genotyping and strain selection

**Fig. 2:**
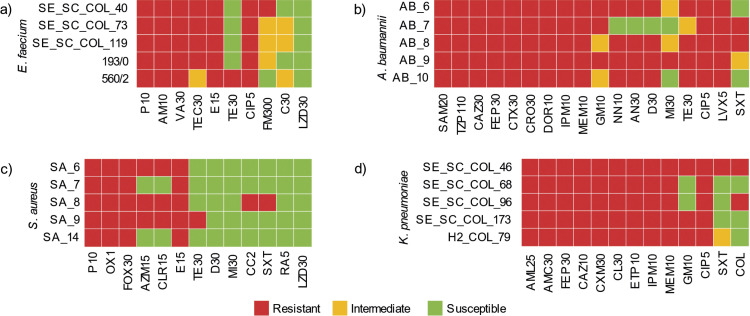
The multidrug-resistant (MDR) phenotype of representative strains: a) *E. faecium*, b) *A. baumannii*, c) *S. aureus* and d) *K. pneumoniae.* Penicillins: P10=penicillin (10 U), AM10=ampicillin (10 µg), OX1=oxacillin (1 µg), AML25=amoxicillin (25 µg); Penicillins+β-lactamase inhibitors: SAM20=ampicillin+sulbactam (10+10 µg), TZP110=piperacillin+tazobactam (100+10 µg), AMC30=amoxicillin+clavulanic acid (20+10 µg); Cephems: CAZ30=ceftazidime (30 µg), CAZ10=ceftazidime (10 µg), FEP30=cefepime (30 µg), CTX30=cefotaxime (30 µg), CRO30=ceftriaxone (30 µg), FOX30=cefoxitin (30 µg), CXM30=cefuroxime (30 µg), CL30=cephalexin (30 µg); Glycopeptides: VA30=vancomycin (30 µg); Lipoglycopeptides: TEC30=teicoplanin (30 µg); Macrolides: E15=erythromycin (15 µg), AMZ15=azithromycin (15 µg), CLR15=clarithromycin (15 µg); Carbapenems: DOR10=doripenem (10 µg), IPM10=imipinem (10 µg), MEM10=meropenem (10 µg), ETP10=ertapenem (10 µg); Aminoglycosides: GM10=gentamicin (10 µg), NN10=tobramycin (10 µg), AN30=amikacin (30 µg); Tetracyclines: TE30=tetracycline (30 µg), D30=doxycycline (30 µg), MI30=minocycline (30 µg); Fluoroquinolones: CIP5=ciprofloxacin (5 µg), LVX5=levofloxacin (5 µg); Nitrofurantoins: FM300=nitrofurantoin (300 µg); Phenicols: C30=chloramphenicol (30 µg); Lincosamides: CC2=clindamycin (2 µg); Folate pathway antagonists: SXT=trimethoprim+sulfomethoxazole (1.25+23.75 µg); Ansamycins: RA5=rifampin (5 µg); Oxazolidinones: LZD30=linezolid (30 µg); Polymyxins: COL=colistin (2 mg/L)

**Fig. S1:**
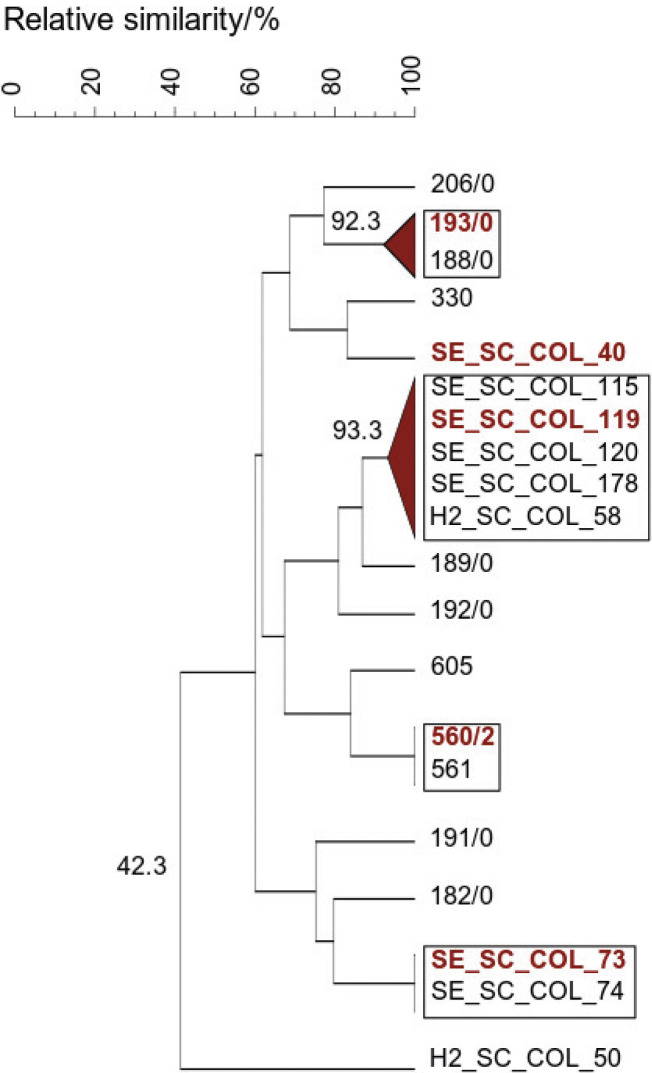
Intraspecies variability of vancomycin-resistant *Enterococcus faecium* (VRE)

**Fig. S2:**
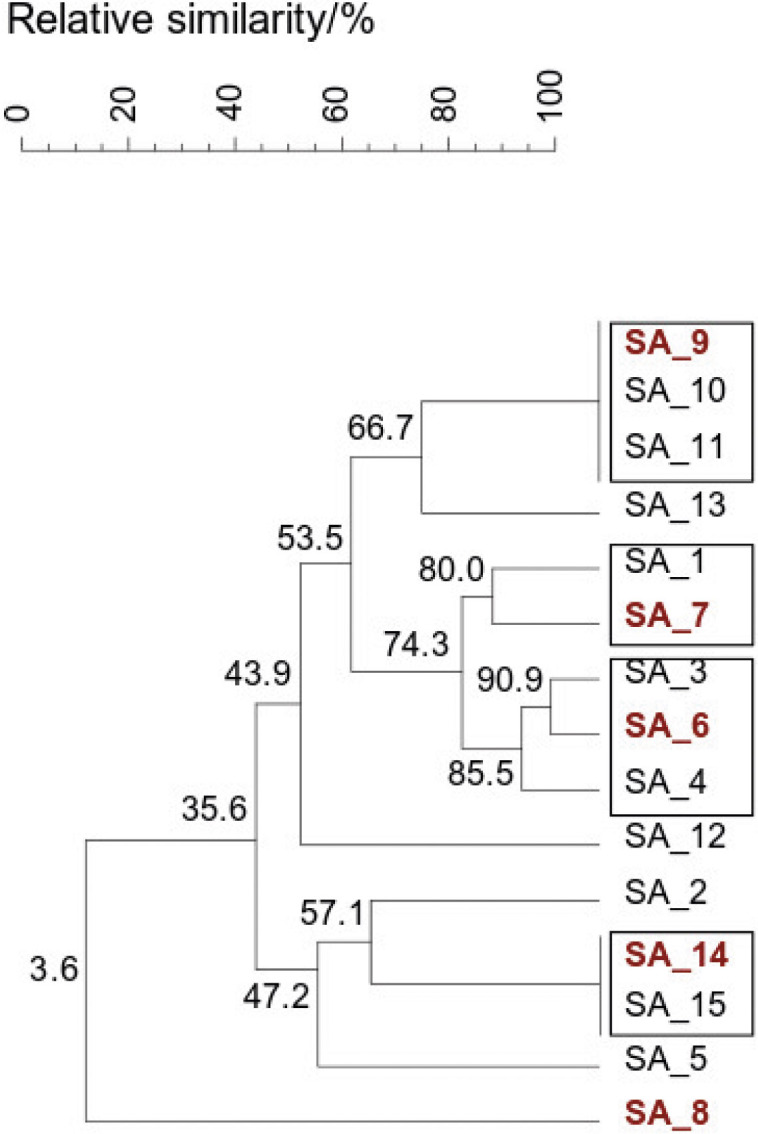
Intraspecies variability of methicillin-resistant *Staphylococcus aureus* (MRSA)

**Fig. S3:**
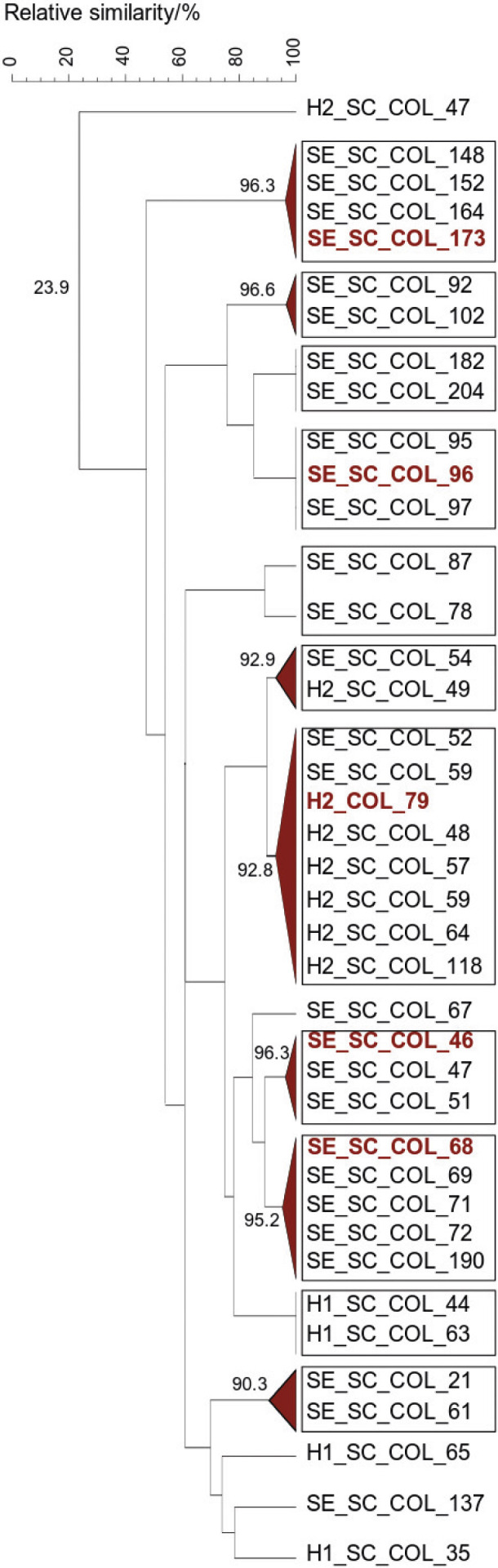
Intraspecies variability of carbapenem-resistant *Klebsiella pneumoniae* (CRKP)

**Fig. S4:**
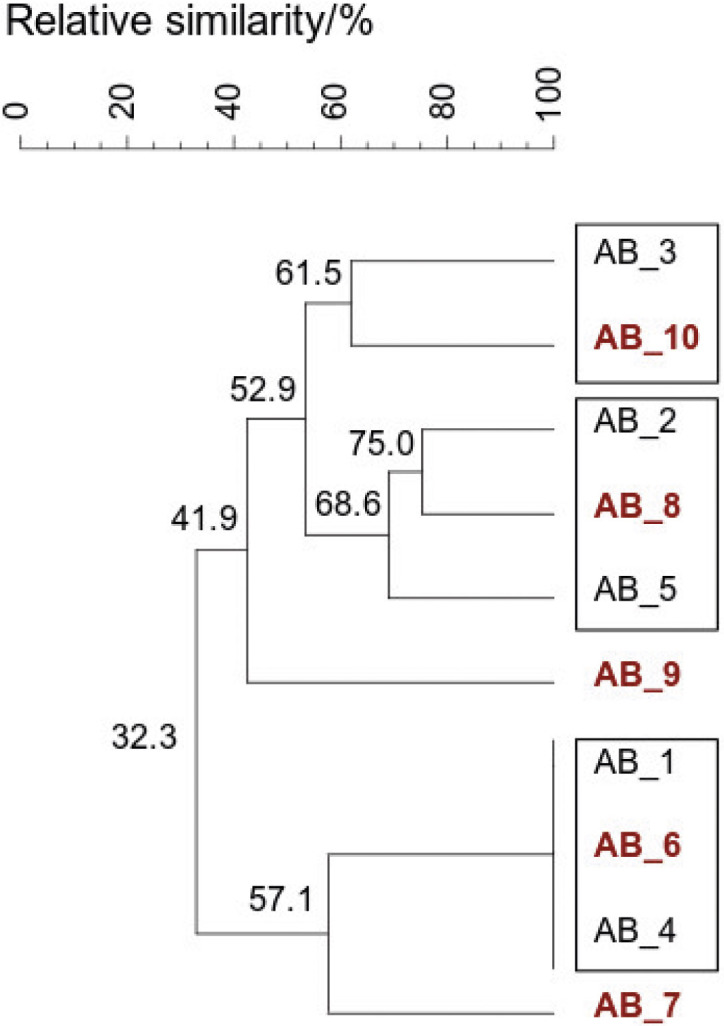
Intraspecies variability of carbapenem-resistant *Acinetobacter baumannii* (CRAB)

All isolates in this study were grouped based on similarity patterns obtained by rep-PCR, which showed a high degree of intraspecies variability. *E. faecium* isolates (*N*=20) were divided into nine unique (monophyletic) profiles and four groups comprising two or more strains (Fig. S1). *S. aureus* isolates (*N*=15) formed five monophyletic clusters and four multi-strain groups (Fig. S2), while *K. pneumoniae* isolates (*N*=40) were grouped into five monophyletic and eleven multi-strain groups (Fig. S3). *A. baumannii* strains (*N*=10) were classified into two monophyletic profiles and three multi-strain groups (Fig. S4). Representative strains (*N*=5) from each species were selected for further analysis based on the following criteria: representatives were primarily chosen from groups containing two or more strains and for species exhibiting higher intraspecies diversity or with fewer available isolates, additional representatives were randomly selected from monophyletic clusters identified in the dendrograms. The multidrug-resistant (MDR) phenotype of the representative strains, confirmed by antibiogram analysis (Fig. 2), revealed carbapenem resistance in *K. pneumoniae* and *A. baumannii*, methicillin resistance in *S. aureus*, and vancomycin resistance in *E. faecium*. The combination of the MDR phenotype and the high virulence potential of these species is a major cause of potentially fatal human infections (*46*), making them bacterial pathogens of significant public health concern (*1*).

### Antibacterial properties of royal jelly samples

The antibacterial activity of the royal jelly samples against MDR pathogenic bacterial strains, assessed by measuring inhibition zone diameters, showed that royal jelly at 1 g/mL exhibited antibacterial activity against the tested bacteria, with inhibition zones ranging from (11.8±2.7) mm for *K. pneumoniae* to (16.8±6.1) mm for *S. aureus* (Table 3). Royal jelly samples from the continental (RJ1 and RJ3) and Mediterranean (RJ4) regions of Croatia inhibited all tested bacteria, while RJ2 and RJ5 inhibited all bacteria except for two MRSA strains (RJ2) and one strain each of *A. baumannii* (RJ5) and *K. pneumoniae* (RJ5) (Table 3).

**Table 3:** The antibacterial activity of royal jelly (RJ1-RJ5) against selected multidrug-resistant (MDR) pathogens as shown by disc diffusion method

Species	Strain	*d*(inhibition zone)/mm				
		RJ1	RJ2	RJ3	RJ4	RJ5
*E. faecium*						
	SE_SC_COL_40	(14.0±0.0)^a^	(11.5±0.7)^b^	(14.0±0.0)^a^	(13.5±0.7)^ab^	(12.0±0.0)^ab^
	SE_SC_COL_73	(15.0±0.0)^a^	(11.5±0.7)^b^	(14.5±0.7)^ab^	(15.0±0.0)^a^	(15.0±0.0)^a^
	SE_SC_COL_119	(16.5±0.7)^ab^	(14.5±0.7)^ab^	(18.5±0.7)^a^	(13.5±0.7)^b^	(13.5±0.7)^b^
	193/0	(14.0±0.0)^ab^	(11.5±0.7)^a^	(15.0±0.0)^b^	(14.5±0.7)^b^	(14.0±0.0)^ab^
	560/2	(12.0±0.0)^ab^	(10.0±0.0)^a^	(13.0±0.0)^ab^	(13.5±0.7)^b^	(13.0±1.4)^ab^
*S. aureus*						
	SA_6	(15.5±0.7)^ab^	(0.0±0.0)^a^	(26.0±0.0)^b^	(26.0±0.0)^b^	(13.0±1.4)^ab^
	SA_7	(16.5±0.7)^ab^	(0.0±0.0)^a^	(17.0±1.4)^b^	(15.5±0.7)^ab^	(17.0±0.0)^b^
	SA_8	(16.0±0.0)^ab^	(15.0±0.0)^a^	(18.5±0.7)^b^	(20.0±1.4)^b^	(17.5±0.7)^ab^
	SA_9	(16.0±1.4)^ab^	(16.5±0.7)^ab^	(20.0±1.4)^a^	(15.5±0.7)^b^	(16.0±0.0)^ab^
	SA_14	(19.5±0.7)^ab^	(16.0±0.0)^a^	(24.5±0.7)^b^	(23.0±0.0)^b^	(19.5±0.7)^ab^
*K. pneumoniae*						
	SE_SC_COL_46	(13.0±0.0)^abc^	(12.0±0.0)^ab^	(15.0±0.0)^c^	(14.0±0.0)^ac^	(11.0±0.0)^b^
	SE_SC_COL_68	(12.0±0.0)^ab^	(10.0±0.0)^a^	(13.0±0.0)^b^	(12.0±0.0)^ab^	(11.5±0.0)^a^
	SE_SC_COL_96	(12.0±0.0)^abc^	(10.0±0.0)^a^	(13.5±0.0)^b^	(12.5±0.0)^bc^	(11.5±0.0)^ac^
	SE_SC_COL_173	(11.5±0.0)^a^	(11.0±0.0)^a^	(14.0±0.0)^b^	(13.5±0.0)^ab^	(13.5±0.0)^ab^
	H2_COL_79	(12.5±0.0)^ab^	(10.5±0.0)^a^	(13.5±0.0)^b^	(12.5±0.0)^ab^	(0.0±0.0)^a^
*A. baumannii*						
	AB_6	(12.0±0.0)^a^	(12.0±0.0)^a^	(16.0±0.0)^ab^	(17.0±0.0)^b^	(14.0±0.0)^ab^
	AB_7	(13.0±0.0)^a^	(13.5±0.0)^ab^	(17.0±0.0)^bc^	(19.5±0.0)^c^	(16.5±0.0)^abc^
	AB_8	(14.0±0.0)^abc^	(10.5±0.0)^ab^	(16.5±0.0)^ac^	(18.0±0.0)^c^	(0.0±0.0)^b^
	AB_9	(14.0±0.0)^ab^	(13.0±0.0)^a^	(16.5±0.0)^b^	(16.5±0.0)^b^	(14.0±0.0)^ab^
	AB_10	(13.0±0.0)^ab^	(12.0±0.0)^a^	(17.0±0.0)^bc^	(18.5±0.0)^c^	(15.5±0.0)^abc^

The inhibition zones are shown as a mean value±standard deviation (*N*=3). Different letters in superscript within a row indicate significant differences in the antibacterial activity of royal jelly based on Dunn's test with Bonferroni adjusted p-values (p<0.05)

To obtain more accurate quantitative results, the well diffusion test was supplemented with the serial microdilution method, as the well diffusion test alone is limited. This is because it relies on uniform diffusion of the tested substance, a property uncommon in most natural compounds, and there is no direct correlation between the antibacterial concentration and inhibition zone size (*47*). Additionally, Osés *et al*. (*48*) noted that the agar well diffusion method has relatively low sensitivity because the tested samples become diluted upon diffusion into the agar. Consistent with these observations, some royal jelly samples did not produce inhibition zones in the agar well diffusion assay but did show antibacterial activity when evaluated by the broth dilution method. Despite minor methodological variations, both approaches confirmed the antibacterial potential of royal jelly and its broad-spectrum activity against MDR Gram-positive and Gram-negative bacteria. However, bacterial susceptibility varied depending on the species and strains, regardless of the method used (Table 3 and Table 4).

**Table 4:** Minimum inhibitory concentration (MIC) and minimum bactericidal concentration (MBC) of royal jelly (RJ1-RJ5) against selected multidrug-resistant (MDR) pathogens

Pathogen	Strain	MIC (MBC)/(µg/mL)				
		RJ1	RJ2	RJ3	RJ4	RJ5
*E. faecium*						
	SE_SC_COL_40	125.0 (125.0)	125.0 (125.0)	62.5 (62.5)	62.5 (62.5)	125.0 (125.0)
	SE_SC_COL_73	125.0 (125.0)	125.0 (125.0)	125.0 (125.0)	62.5 (125.0)	62.5 (125.0)
	SE_SC_COL_119	31.3 (31.3)	125.0 (125.0)	62.5 (125.0)	125.0 (125.0)	31.3 (62.5)
	193/0	125.0 (125.0)	125.0 (250.0)	62.5 (62.5)	62.5 (125.0)	125.0 (125.0)
	560/2	125.0 (250.0)	250.0 (250.0)	125.0 (250.0)	250.0 (250.0)	250.0 (250.0)
*S. aureus*						
	SA_6	15.6 (15.6)	62.5 (62.5)	31.3 (31.3)	31.3 (31.3)	31.3 (31.3)
	SA_7	15.6 (15.6)	31.3 (31.3)	31.3 (31.3)	15.6 (15.6)	15.6 (15.6)
	SA_8	62.5 (62.5)	62.5 (62.5)	62.5 (62.5)	62.5 (62.5)	62.5 (62.5)
	SA_9	31.3 (31.3)	62.5 (62.5)	31.3 (31.3)	31.3 (31.3)	31.3 (31.3)
	SA_14	15.6 (15.6)	31.3 (31.3)	31.3 (31.3)	15.6 (31.3)	15.6 (31.3)
	SE_SC_COL_46	125.0 (125.0)	500.0 (500.0)	62.5 (62.5)	125.0 (125.0)	62.5 (62.5)
	SE_SC_COL_68	125.0 (125.0)	250.0 (250.0)	62.5 (62.5)	15.6 (15.6)	62.5 (62.5)
	SE_SC_COL_96	125.0 (125.0)	125.0 (125.0)	62.5 (62.5)	62.5 (62.5)	125.0 (125.0)
	SE_SC_COL_173	125.0 (125.0)	125.0 (125.0)	62.5 (62.5)	125.0 (125.0)	62.5 (62.5)
	H2_COL_79	125.0 (125.0)	250.0 (250.0)	62.5 (62.5)	62.5 (62.5)	125.0 (125.0)
*A. baumannii*						
	AB_6	62.5 (62.5)	62.5 (62.5)	31.3 (31.3)	31.3 (31.3)	31.3 (31.3)
	AB_7	15.6 (15.6)	31.3 (31.3)	15.6 (15.6)	15.6 (15.6)	31.3 (31.3)
	AB_8	15.6 (15.6)	7.8 (7.8)	15.6 (15.6)	15.6 (15.6)	31.3 (31.3)
	AB_9	15.6 (15.6)	62.5 (62.5)	15.6 (15.6)	15.6 (15.6)	31.3 (31.3)
	AB_10	15.6 (15.6)	31.3 (31.3)	31.3 (31.3)	31.3 (31.3)	15.6 (15.6)

Values in the parentheses are the MBC values

Notably, *S. aureus* and *A. baumannii* showed significantly higher susceptibility to royal jelly, while *E. faecium* and *K. pneumoniae* showed the lowest susceptibility (Table 4). The antibacterial properties of royal jelly are attributed to its bioactive compounds, including proteins, peptides, phenolic compounds and fatty acids (*49*). Certain constituents, such as royalisin, have stronger activity against Gram-positive bacteria (*50*), while most bioactive compounds exert broad-spectrum, non-selective effects on bacterial cells, mainly by disrupting membranes, lysing intracellular contents, or inhibiting the synthesis of various essential cellular components, transcription and translation (*51*, *52*). Although the peptidoglycan layer is not an effective permeability barrier in Gram-positive bacteria compared to the outer membrane of Gram-negative bacteria (*5*), no significant differences between Gram-positive and Gram-negative bacteria in terms of inhibition zone diameter and MICs were observed in our study (Table 5). A significant effect was found only for MBC values, indicating higher susceptibility of Gram-negative bacteria, which contradicts previous findings (*5*, *50*). Interestingly, the Gram-negative *A. baumannii* was highly susceptible to royal jelly (MIC/MBC (27.2±15.5) µg/mL), regardless of the strain tested, while Gram-positive *E. faecium* strains showed the highest resistance (MIC/MBC=(96.6±36.0)/(126.4±58.2) µg/mL) among the four species tested.

**Table 5:** The effect of species, Gram staining, and royal jelly origin on antibacterial activity of royal jelly against selected multidrug-resistant (MDR) pathogens

Factor	*d*(inhibition zone)/mm	MIC/(µg/mL)	MBC/(µg/mL)
Species			
*E. faecium*	(13.7±1.8)^a^	(96.6±36.0)^a^	(126.4±58.2)^a^
*S. aureus*	(16.8±6.1)^b^	(35.6±18.3)^b^	(36.9±17.4)^b^
*K. pneumoniae*	(11.8±2.7)^c^	(91.6±35.5)^a^	(91.6±35.5)^a^
*A. baumannii*	(14.4±3.8)^a^	(27.2±15.5)^b^	(27.2±15.5)^b^
Gram staining			
Positive	(15.3±4.7)^a^	(57.3±41.9)^a^	(78.8±61.2)^a^
Negative	(13.1±3.5)^a^	(64.2±41.4)^a^	(57.3±41.9)^b^
Royal jelly			
RJ1	(14.1±2.1)^a^	(71.1±51.8)^a^	(77.3±64.6)^a^
RJ2	(11.1±4.2)^a^	(74.7±43.2)^a^	(82.5±60.6)^a^
RJ3	(16.7±3.5)^a^	(52.3±31.0)^a^	(61.7±54.0)^a^
RJ4	(16.2±3.7)^a^	(51.0±38.2)^a^	(58.4±44.1)^a^
RJ5	(12.9±4.9)^a^	(56.7±39.7)^a^	(62.5±41.3)^a^
Origin			
Continental	(13.9±4.1)^a^	(65.4±43.2)^a^	(73.2±59.4)^a^
Dalmatia	(14.6±4.6)^a^	(53.9±38.6)^a^	(60.4±42.2)^a^

The growth inhibition zone, minimum inhibitory (MIC) and minimum bacterial concentration (MBC) are shown as mean value±standard deviation. Different letters in superscript within a column indicate significant differences in the antibacterial activity of royal jelly for each factor based on Dunn's test with Bonferroni adjusted p-values (p<0.05)

Additionally, the antibacterial activity of royal jelly against certain MDR pathogens varied not only between different bacterial species, but also among different strains within the same species. For example, *K. pneumoniae* strain SE_SC_COL_68 was inhibited at a concentration of 15.6 µg/mL, while a concentration of 500 µg/mL was required to inhibit strain SE_SC_COL_46. Similarly, *A. baumannii* strains exhibited MIC/MBC values between 7.8 and 62.5 µg/mL, while *E. faecium* strains had MIC/MBC values between 31.3 and 250 µg/mL. The most consistent antibacterial effect was observed against *S. aureus*, with MIC values for the tested strains ranging from 15.6 to 62.5 µg/mL (Table 4).

In general, the MIC and MBC values required to suppress the growth of MDR pathogens in this study were between 7.8 and 500.0 µg/mL (Table 4). These values indicate significantly stronger antibacterial activity than reported in most previous studies testing various royal jelly samples against non-MDR strains of *K. pneumoniae*, *S. aureus* and *E. faecium*, where effective concentrations ranged from 3.7 to 14.5 mg/mL (*3*). Similar to our results, Moselhy *et al*. (*53*) reported comparable MIC/MBC values of royal jelly against non-MDR *Bacillus cereus* and *S. aureus*, ranging from 7.8 to 500.0 µg/mL and 15.6 to 500.0 µg/mL, respectively. However, only a few studies have specifically investigated the activity of royal jelly against MDR bacteria. For example, Uthaibutra *et al*. (*5*) observed MIC/MBC values between 37.5 and 75 mg/mL for royal jelly against methicillin-resistant *S. aureus.* When jelleine I, a peptide derived from royal jelly, was used to inhibit the growth of carbapenem-resistant *A. baumannii* (*54*) and MRSA (*55*), much lower concentrations were required: between 0.32 and 0.6 µg/mL and 8 and 138 µg/mL, respectively. However, to the best of our knowledge, the effect of royal jelly or its individual components on different MDR strains within the same bacterial species has not yet been investigated.

Consistent with the chemical profiling, no significant differences in the antibacterial efficacy of royal jelly samples from different geographical regions of Croatia (Mediterranean *vs* continental) were observed (Table 5), despite considerable variation in their chemical composition. Nevertheless, all royal jelly samples demonstrated consistent antibacterial activity. This suggests a stable and reliable antibacterial profile, likely influenced by local microclimatic conditions rather than broader regional factors. Furthermore, our study supports the hypothesis of a synergistic effect of crude royal jelly against MDR pathogens, as no significant differences in antibacterial activity were observed among the samples, although the amounts of certain bioactive compounds (*e.g.* proteins, lipids and octanoic acid) were higher in RJ1. This concordance is an important aspect for the potential therapeutic use of royal jelly, especially in the treatment of bacterial MDR infections.

## CONCLUSIONS

This study highlights the broad-spectrum antibacterial activity of royal jelly (RJ) against several strains of clinically relevant Gram-positive and Gram-negative multidrug-resistant (MDR) pathogens, including vancomycin-resistant *Enterococcus faecium*, methicillin-resistant *Staphylococcus aureus*, carbapenem-resistant *Klebsiella pneumoniae* and *Acinetobacter baumannii*. Although some royal jelly samples contained higher amounts of bioactive compounds, their superior chemical profile did not consistently result in greater antimicrobial efficacy, regardless of regional origin (Mediterranean *vs* continental Croatia). This observation may indicate that the antibacterial activity of crude royal jelly arises from complex interactions among its components rather than from individual compounds alone, a hypothesis that requires further targeted investigation. In addition to variability between species, significant differences in antimicrobial activity at the strain level were also observed.

To the best of our knowledge, this is the first study to investigate antibacterial effect of royal jelly against multiple MDR strains while simultaneously providing a comprehensive chemical characterization. Our results show for the first time that the efficacy of royal jelly varies not only between species but also between strains of the same species, emphasizing the need to consider both chemical composition and intraspecies diversity of pathogens when assessing its therapeutic potential.

## SUPPLEMENTARY MATERIAL

Supplementary materials are available at: www.ftb.com.hr.

## Appendix

**Table S1:** The list of antibiotics used in this study to validate the multidrug-resistant (MDR) profiles of *E. faecium*, *A. baumannii*, *S. aureus* and *K. pneumoniae* strains

Species	Class of antibiotics	Antibiotic	Abbreviation	Disc content
*E. faecium*	Penicillins	Penicillin	P10	10 U
				*m*/µg
		Ampicillin	AM10	10
	Glycopeptides	Vancomycin	VA30	30
	Lipoglycopeptides	Teicoplanin	TEC30	30
	Macrolides	Erythromycin	E15	15
	Tetracyclines	Tetracycline	TE30	30
	Fluoroquinolones	Ciprofloxacin	CIP5	5
	Nitrofurantoins	Nitrofurantoin	FM300	300
	Phenicols	Chloramphenicol	C30	30
	Oxazolidinones	Linezolid	LZD30	30
*A. baumannii*	Penicillins+β-lactamase inhibitors	Ampicillin+sulbactam	SAM20	10+10
		Piperacillin+tazobactam	TZP110	100+10
	Cephems	Ceftazidime	CAZ30	30
		Cefepime	FEP30	30
		Cefotaxime	CTX30	30
		Ceftriaxone	CRO30	30
	Carbapenem	Doripenem	DOR10	10
		Imipinem	IPM10	10
		Meropenem	MEM10	10
	Aminoglycosides	Gentamicin	GM10	10
		Tobramycin	NN10	10
		Amikacin	AN30	30
	Tetracyclines	Doxycycline	D30	30
		Minocycline	MI30	30
		Tetracycline	TE30	30
	Fluoroquinolones	Ciprofloxacin	CIP5	5
		Levofloxacin	LVX5	5
	Folate pathway antagonists	Trimethoprim+sulfomethoxazole	SXT	1.25+23.75
*S. aureus*	Penicillins	Penicillin	P10	10 U
				*m*/µg
		Oxacillin	OX1	1
	Cephems	Cefoxitin	FOX30	30
	Macrolides	Azithromycin	AZM15	15
		Clarithromycin	CLR15	15
		Erythromycin	E15	15
	Tetracyclines	Tetracycline	TE30	30
		Doxycycline	D30	30
		Minocycline	MI30	30
	Lincosamides	Clindamycin	CC2	2
	Folate pathway antagonists	Trimethoprim+sulfomethoxazole	SXT	1.25+23.75
	Ansamycins	Rifampin	RA5	5
	Oxazolidinones	Linezolid	LZD30	30
*K. pneumoniae*	Penicillins	Amoxicillin	AML25	25
	Penicillins+β-lactamase inhibitors	Amoxicillin+clavulanic acid	AMC30	20+10
	Cephems	Cefepime	FEP30	30
		Ceftazidime	CAZ10	10
		Cefuroxime	CXM30	30
		Cephalexin	CL30	30
	Carbapenem	Ertapenem	ETP10	10
		Imipenem	IPM10	10
		Meropenem	MEM10	10
	Aminoglycosides	Gentamicin	GM10	10
	Fluoroquinolones	Ciprofloxacin	CIP5	5
	Folate pathway antagonists	Trimethoprim+sulfamethoxazole	SXT	1.25+23.75
				*γ*/(mg/L)
	Polymyxins	Colistin	COL	2
